# Modeling Psychological Attributes in Psychology – An Epistemological Discussion: Network Analysis vs. Latent Variables

**DOI:** 10.3389/fpsyg.2017.00798

**Published:** 2017-05-18

**Authors:** Hervé Guyon, Bruno Falissard, Jean-Luc Kop

**Affiliations:** ^1^INSERM U1018, CESP, APHP, Université Paris-Sud, UVSQ, Université Paris-SaclayVillejuif, France; ^2^IUT de Sceaux – Université Paris-SudSceaux, France; ^3^Laboratoire Interpsy – 2LPN (CEMA), Université de LorraineNancy, France

**Keywords:** Latent Variables, Network Analysis, complex systems, pragmatism-realism, psychological attributes, epistemology in psychology, Latent Network models

## Abstract

Network Analysis is considered as a new method that challenges Latent Variable models in inferring psychological attributes. With Network Analysis, psychological attributes are derived from a complex system of components without the need to call on any latent variables. But the ontological status of psychological attributes is not adequately defined with Network Analysis, because a psychological attribute is both a complex system and a property emerging from this complex system. The aim of this article is to reappraise the legitimacy of latent variable models by engaging in an ontological and epistemological discussion on psychological attributes. Psychological attributes relate to the mental equilibrium of individuals embedded in their social interactions, as robust attractors within complex dynamic processes with emergent properties, distinct from physical entities located in precise areas of the brain. Latent variables thus possess legitimacy, because the emergent properties can be conceptualized and analyzed on the sole basis of their manifestations, without exploring the upstream complex system. However, in opposition with the usual Latent Variable models, this article is in favor of the integration of a dynamic system of manifestations. Latent Variables models and Network Analysis thus appear as complementary approaches. New approaches combining *Latent Network Models* and *Network Residuals* are certainly a promising new way to infer psychological attributes, placing psychological attributes in an inter-subjective dynamic approach. Pragmatism-realism appears as the epistemological framework required if we are to use latent variables as representations of psychological attributes.

## Introduction

Latent Variable models are commonly used in psychological research to model the measurements of psychological attributes and causal relations between these measures (Bollen, 1989; Kelava and Brandt, 2014). However, the validity of these models has been challenged in the academic literature, and these criticisms encouraged Markus and Borsboom (2013) to discuss the legitimacy of the use of latent variables in psychological research. In their book, it is suggested that recent approaches, such as Network Analysis, are potentially more efficient for the study of psychological attributes, because they are closer to reality than conventional approaches entailing latent variables (Borsboom and Cramer, 2013; De Schryver et al., 2015).

Network Analysis does appear to possess real interest in psychopathology (Bringmann et al., 2015; Fried et al., 2017). But beyond methodological aspects, it raises a more general epistemological issue for psychology (Dalege et al., 2016; Borsboom, 2017). With Network Analysis, a psychological attribute is not considered as an underlying common cause that explains perceptible manifestations. Here, a psychological attribute is a complex system of perceptible components^1^, i.e., a system in which each component interacts with every other without these perceptible components being linked to an underlying common cause (Cramer et al., 2010, 2012a; Borsboom and Cramer, 2013; Bringmann et al., 2013; Schmittmann et al., 2013; De Schryver et al., 2015; Fried, 2015; McNally et al., 2015; Dalege et al., 2016). Abandoning latent variables raises the issue of the ontology of psychological attributes.

We agree with the criticism of an essentialist epistemology in the area of psychological attributes (Fried, 2015). We agree with Zachar (2010) that psychology must break with the dominant epistemology of realism involving latent variables, “biological realism” (Lloyd, 2010), which considers a psychological attribute as an entity in the brain. But this does not mean that we must return to an instrumentalist/constructivist epistemology, as Network Analysis appears to do (Zachar, 2010; Fried et al., 2016). We consider that in psychology we need to adopt a pragmatist and realist epistemology (Putnam, 1981, 1992; Maul, 2013). This could provide a clarification of the ontological status of psychological attributes: a psychological attribute is not an entity in the brain independent from the individual embedded in social interactions.

The aim of this article is to reappraise the legitimacy of Latent Variable models by way of an ontological and epistemological discussion on psychological attributes. The structure of this paper is as follows. A first section concerns Network Analysis and contradictions among authors who advocate Network Analysis against Latent Variable models. In the second section, we explain why a pragmatist and realist epistemology is required to avoid the issue of the ontology of psychological attributes. The third section will show that the Latent Variable model is an efficient approach to infer psychological attributes within a pragmatist-realist epistemology. Finally, in the fourth section we will discuss the complementary nature of the two approaches (Network Analysis and Latent Variable models), and the epistemological implications. The discussion uses an example based on the construct of *depression*.

## Network Analysis: Contributions and Contradictions

Recently, various authors have proposed Network Analysis as an alternative approach to the classic Latent Variable approach. If Network Analysis can avoid some problems that are inherent in Latent Variable approaches, we consider that it also involves contradictions. Because the Network Analysis literature is recent and certainly not familiar to some readers, we back up our explanations with several passages quoted from authors who advocate Network Analysis.

### Latent Variables and Network Analysis

In this paper we consider that psychological attributes are psychological properties of an individual (Markus and Borsboom, 2013). Most often, tests or scales are used to measure^2^ these psychological attributes, with scores that estimate underlying latent variables. For readers seeking a reference on the notion of the latent variable in psychometrics, see Bollen (1989, 2002) and Borsboom et al. (2003).

**FIGURE 1:**
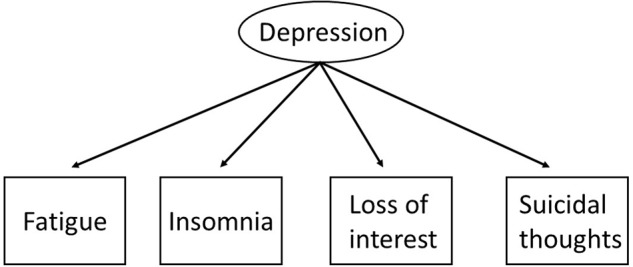
**An illustration of Depressionusing, a Latent Variable model**.

For example, we can consider a depression scale with four items that correspond to certain manifestations of the disorder (**Figure 1**). Within this framework, the covariance of these items can be explained by the common influence of a latent variable (Fried et al., 2016): in other words the items are manifestations of a particular underlying attribute.

For the authors who developed Network Analysis approach, there is a fundamental difference between the Latent Variable approach and Network Analysis. For these authors, with Network Analysis, we do not observe/measure “manifestations” of an underlying attribute. It is the network of relationships between the items (here referred to as components) that is considered to constitute the psychological attribute (De Schryver et al., 2015). Psychological attributes, according to this view, exist as *systems* where components mutually influence each other without the need to call on latent variables (Cramer et al., 2012a; Schmittmann et al., 2013). These authors thus consider that there is no underlying cause within the relationship between the components (Cramer et al., 2010; Borsboom and Cramer, 2013; Borsboom, 2017). The Network approach thus does away with the notion of the latent variable (Cramer et al., 2010; Nuijten et al., 2016). From a statistical point of view, the local independence assumption, inherent in the Latent Variable approach, is consequently no longer necessary.

**FIGURE 2:**
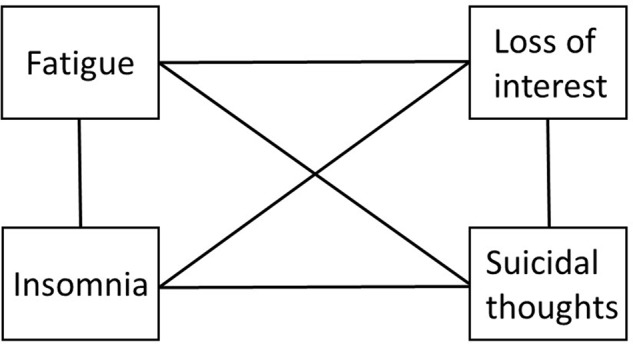
**An illustration of Depression using a Network Analysis model**.

Returning to our example concerning depression, a Network model of the four measured components could be represented as in **Figure 2** (Fried et al., 2015).

Considering a psychological attribute as a network of components raises certain issues. With Network Analysis, the psychological attribute is “something real… but what?” (Borsboom and Cramer, 2013, p. 115). This “what?” raises the question of the ontology of psychological attributes.

### Attractors and Network Analysis

In the literature on the Network Analysis approach in psychometrics, there is a gray area concerning the ontology of psychological attributes. In these articles, a psychological attribute is considered as a complex network of components and at the same time as a state of equilibrium in an individual (the emphasis is ours): “For some reason, human systems tend to settle in relatively fixed areas of the enormous behavioral space at their disposal, where they are in relative ‘equilibrium’ with themselves and their environments” (Cramer et al., 2012a, p. 416). A connection is made by a number of authors between the dynamic complex system and this state of equilibrium, by considering that this state is an *attractor* within the complex dynamic system (in the following, the emphasis is ours): “This definition of equilibrium is analogous to ‘attractors’ in the complex systems literature” (Cramer et al., 2012a, p. 416); “Which attractor states exist in an attitude network likely depends on the connectivity of the network” (Dalege et al., 2016, p. 17); “If the system is close to an attractor state, it will converge to it, and remain in there in equilibrium” (Schmittmann et al., 2013, p. 47); “it will encounter one or more attractors in state space: regions in state space that the system will move toward and enter. In state spaces with more than one attractor, some systems tend to move toward one attractor and remain there in a stable state” (Cramer et al., 2010, p. 149). “The notion of mental health may be defined as the stable state of a weakly connected network… A mental disorder itself assumes a new definition as the (alternative) stable state of a strongly connected network” (Borsboom, 2017, p. 9). “We can use the network of Susan as an example of a bi-stable system with two attractor states: a healthy and a sick state” (Fried et al., 2017, p. 4). “MD specifically is hypothesized to be a bistable system with two attractor states: a ‘non-depressed’ and a ‘depressed’ state […] Network models from other areas of science show that strong connections between elements of a dynamic system predict the tipping of that same system from one attractor state into another” (Cramer et al., 2016, p. 2).

Thus, in the Network Analysis approach, even if psychological attributes are presented as Network of components, in fact the Network Analysis literature considers them as states of equilibrium because dynamic systems of components generate a particularly stable organization of the system: an attractor within the network.

### Emergent Properties and Network Analysis

The ontology of an attractor from a complex system is generally defined in relation to the concept of *emergence* (Humphreys, 2008). An attractor is an emergent property because of the new structures and functions that are emerging, which means that the new property is not reducible to the properties of the basic elements, and not logically predictable from the basic elements (Barrett, 2011; Maul, 2013).

In the Network Analysis literature, few authors have explicitly related *attractors* to *emergence*, but they use wordings that suggests this relationship. For example (in the following, the emphasis is ours), Cramer et al. (2010, p. 149) explicitly link Network Analysis to emergent properties: “In complexity research, rapid advances are made with respect to modeling emerging properties in complex systems, and the network approach for mental disorders could benefit from those advances.” Cramer et al. (2012a, p. 418) noted: “Thus, we can still use a term such as ‘neuroticism’ to refer to a phenomenon that emerges as a result of the biological, psychological, and environmental forces that knit some behaviors closely together.” Another example from Cramer et al. (2012b, p. 453) is: “Personality traits emerge out of the interactions between personality components.” Also, Schmittmann et al. (2013, p. 48) concluded: “Therefore, if we allow these parameters to change, the system may show qualitative changes in its structure (e.g., a new attractor emerges).” As Cramer et al. (2012a, p. 429) consider: “The network perspective takes the best of both worlds: it can explain how traits emerge out of the network structure”.

In psychology, the emergent property generally derives from a complex system of neurons (Fingelkurts and Fingelkurts, 2004; Barrett, 2009, 2011; Van Orden et al., 2011; Fingelkurts et al., 2013). With Network Analysis, emergence derives from a complex system of components. But the epistemological issue is the same: emergence is related to a new property that is not reducible to the basic elements of the system (components, with Network Analysis).

### Contradictions with Network Analysis

Methodologically, the question raised is how to assess this emergent property. In the articles that advocate the Network Analysis approach in psychometrics, the complex system is assumed to be characterized by a simulated network based on empirical data. Concretely, “human cognition is simulated with a network of several interrelated nodes” (Dalege et al., 2016, p. 3). The nodes are the components (or symptoms) of the psychological attribute under study. This model “deal[s] with complex systems,” and at the same time can be “simulated” using empirical data (Dalege et al., 2016, p. 2). Consequently, for these authors, network is “a realistic conceptualization of empirical data on attitudes” (Dalege et al., 2016, p. 16) and “empirical data would be to use empirically estimated attitude networks as input for data simulation” (Dalege et al., 2016, p. 16). In a similar manner, Cramer et al. (2012a, p. 418) consider that “by studying correlations and representing them in a network structure, one may obtain a first glance at the visualization of the global (i.e., average) structure of personality components”.

However, as explained above, if a psychological attribute is considered as an attractor with an emergent property arising within the complex dynamic system of components, it means that the emergent property is different from and not reducible to the network. Consequently, the use of Network Analysis to infer psychological attributes contradicts this particular conception of psychological attributes, because it is focused on the network itself and does not therefore provide a way to assess emergent properties.

## Psychological Attributes in Pragmatist-Realist Epistemology

Psychological attributes do not exist as entities independent from human perceptions, but they *do* correspond to some kind of reality. This “in-between” position is often related to a specific epistemological pragmatist and realist framework (Putnam, 1981, 1992; Baggini and Fosl, 2010; Maul, 2013).

### The Ontology of Psychological Attributes

Among authors who advocate Network Analysis a major reason for not linking components to a latent psychological attribute is the absence of clear-cut brain structures, each corresponding to specific psychological attribute. For Zachar (2010), Network Analysis highlights the mistaken ontology of psychological attributes that dominates psychology. Zachar (2010) considers that Network Analysis tends to have an anti-realist point of view.

“Reality” does not necessarily correspond here to a separate entity in the brain, as Cramer et al. (2010) and Borsboom and Cramer (2013) seem to suggest. Psychological attributes can be considered as robust attractors within a complex dynamic arising from mental processes with emergent properties (Fingelkurts and Fingelkurts, 2004; Barrett, 2009, 2011; Van Orden et al., 2011; Fingelkurts et al., 2013; Maul, 2013). When psychological attributes are defined as attractors, it is the ontology of psychological attributes that is reassessed. Because psychological attributes are linked to the mental equilibrium of an individual, they should be considered as realities, as states of equilibrium of individuals in their social interactions (Van Geert and Steenbeek, 2010).

This “equilibrium” needs to be analyzed in a dynamic relationship with the social environment (Millikan, 1995). The brain has a specific stable organization, which is an attractor, although it is not possible to describe it precisely. First, an attractor is a non-predictable specific organization of a system, so that it is impossible to define it more precisely in the brain (Barrett, 2009). Second, this attractor is dependent on the environment (Millikan, 1995; Cervone, 2010). The materiality of the psychological attribute is not a complex system but an emergent property of the individual, it is therefore a materiality embedded in social interactions. Indeed, a psychological attribute exists as a function in our social relations. This materiality is therefore both objective (the complex process generating emergent property) and intersubjective (the way in which emergent property is conceptualized in a particular social space). Rather than considering that psychological attributes need to be locally defined by a specific entity in the brain, it is possible to consider psychological attributes as realities, overcoming this requirement (Maul, 2013).

### Pragmatism-Realism

The central pragmatist author Dewey (1998) held that a psychological attribute results from transactions by individuals with their social and biological environment. This holistic and embedded point of view is close to that of Varela et al. (1992) who explicitly linked the emergence of psychological attributes to a pragmatist epistemology. The embodied approach, or *embodiment* (Lloyd, 2010), is gaining ground in the field of psychology (Willems and Francken, 2012; Harris et al., 2015; Soylu, 2016). Because scientific theory is related to abstract cognition (Dijkstra et al., 2014), embodiment also entails a scientific approach. Pragmatic epistemology is an epistemology that considers that scientific theory is influenced by context, culture, etc., and cannot be considered independently from this practical engagement with the world (Allen and Clough, 2015).

This does not mean that we have to relinquish realism, rather that we need to adopt a form of pragmatism-realism, as suggested by Putnam (1981), Maul (2013) and Allen and Clough (2015) for example. Realistic pragmatism is a practical framework of knowledge in psychology that is opposed to skepticism toward objective knowledge. Every theory is contingent on objectives, but dependent on reality. Realism remains the background condition for all intelligibility (Searle, 1996). Pragmatism-realism makes it possible to avoid an epistemological framework that entails a purely instrumentalist and therefore relativistic science. Realism that considers that the psychological attributes analyzed in psychology are not fixed and not external to our social praxis, and that psychological attributes relate both to reality and to a social construction, can be considered as both pragmatist and realist (Putnam, 1981, 1992; Maul, 2013).

Putnam (1981, 1992), often considered as central in the new-pragmatism epistemology, considers that a pragmatist epistemology of this sort is not against realism, but is a “realism for us”. Pragmatism-realism clearly enables a psychological attribute to be considered as a real attribute of the individual embedded in social praxis, an attribute of behavior, the conceptualization of which is consequently also embedded in a social praxis.

For us, pragmatism is a method rather than a philosophy in the strict sense. It distinguishes itself from a static conception of reason; it privileges processes and approaches without entering into an integral relativism. Since pragmatism does not constitute a homogeneous current, and in order to differentiate ourselves from a pragmatism close to relativism, we will refer to realism-pragmatism to characterize the epistemology that we consider should be used. But clearly, like Hacking (2007), we are pragmatic with pragmatism, and we restrict the pragmatist-realist method proposed here to the ontology of psychological attributes, without positioning ourselves firmly and definitively in any particular *-ism*.

### Categories

The classification of psychological attributes raises a central issue between *essentialism* and *nominalism* (Kukla, 2001; Zachar and Kendler, 2007). *Essentialism* is the view that psychological attributes have a well-defined hidden nature; and because the psychological attribute exists independent of our classifications, categories formalize this underlying nature (Kukla, 2001; Zachar and Kendler, 2007). *Nominalism* is the view that psychological attributes are constructed categories without natural referent, merely practical categories for particular uses (Kukla, 2001; Zachar and Kendler, 2007).

We consider that the opposition between realism and instrumentalism/constructivism can be overcome by a pragmatist-realist position (Putnam, 1981, 1992). A concept in psychology can be considered as referring neither to a fixed reality (essentialist view), nor to a singular construction independent from reality (nominalist view). Hacking (2000) considered that social constructions and reality seem to be mutually exclusive (realism vs. instrumentalism/constructivism); but part of the tension between the two results from the interactions between them. This is not a skeptical view of the categories of psychological attributes. Psychological categories are derived from human experience and social interactions, and are linked to realities (Moore, 1985). But, psychological concepts relate to objects that are defined as *ontologically subjective* by Searle (1996) because the conceptualization of a psychological attribute is *observer-dependent* (Barrett, 2009). Psychological attributes exist because they are perceptible manifestations. In the words of Searle: “mental states are distinguished from other physical phenomena in that they are either conscious or potentially so. Where there is no accessibility to consciousness, at least in principle, there are no mental states” (Searle, 1996, p. 228).

We consider that a psychological attribute is a reality because it exists as a social praxis. Categorization in psychology is consequently different from what it is in physics (Hacking, 2000). Psychological attribute categories are defined in relation to human goals rather simply discovered as entities existing in the world (Zachar and Kendler, 2007). Categories used in psychology are derived from inter-subjective factors (Allen and Williams, 2011; Markon, 2013, 2015). As with Zachar’s (2002) proposal for a pragmatic procedure to categorize psychological attributes, the fact that there are practical models to categorize psychological attributes does not deny that psychological attributes are based on realities. It only denies that psychological categories are solely determined by natural categories because psychological attributes are constructed as well as discovered (Zachar, 2002; Zachar and Kendler, 2007). Psychological categories should be considered as relational categories, *interactive genres* (Hacking, 2000). These interactive genres assume that categories are always relative to a social practice, and above all relative to the social praxis of the experts that develop concepts and theories. Because the categorization of psychological attributes involves interaction between reality and social constructions, for Hacking (2000) these categories developed by experts are moving categories. The history of the *Diagnostic and Statistical Manual of Mental Disorders* (DSM) classification is an example of this liability to evolve (see Markon, 2013).

### The Example of Depression

*Depression* illustrates this tension between social construction and reality and also illustrates our pragmatist-realist point of view. There is a large corpus in the literature on Network Analysis and *Depression* (Fried et al., 2014, 2015, 2016; Bringmann et al., 2015; Fried, 2015; Fried and Nesse, 2015; Boschloo et al., 2016; Cramer et al., 2016). The essentialist model, which considers *Depression* as a unique underlying biological entity conceptualized in an undifferentiated category, is certainly criticized because *Depression* refers to mental disorders that are heterogeneous (see Fried, 2015; Fried and Nesse, 2015; Fried et al., 2016). We are in agreement with Kendler and other authors in considering that *Depression* is not a natural psychological attribute independent from social connections (Kendler et al., 2011). *Depression* is a practical category based on social considerations, and *Depression* has been considered in different ways in the past and today (Beck, 1961; Faravelli et al., 1996; Aggen et al., 2005; Norton et al., 2013; Beck and Bredemeier, 2016).

However, we cannot consider that *Depression* is solely a social construct without links with a form of reality. Hippocrates and other ancient Greeks used the term *Melancholia* in reference to a psychological attribute of an individual close (but not identical) to what we today call *Depression*. While we do not categorize and understand *Depression* as the ancient Greeks did with *Melancholia*, we cannot consider that there is nothing in common between the earlier *Melancholia* and the more modern *Depression*. As Hacking suggests, we should consider *Depression* in an interactive way between a reality and the social integration of this reality. There is therefore a *loop effect*, as defined by Hacking (2000). Depression is not a natural category, it is a reified category based on social manifestations. This reified psychological attribute appears as a reality to individuals suffering from depression, and to their social environment. Depression causes changes in individuals because these individuals will integrate their depressive state and behave (partly) in accordance with what society refers to as “being depressed”; likewise, the social environment of the individual adapts its behaviors and attitudes to what society considers to be appropriate with a depressed individual. This is a pragmatist-realist view of Depression. It relates to an underlying mental mechanism in an individual. These underlying mechanisms are certainly different for different individuals categorized as depressive. But the materiality of *Depression* for an individual is not this complex mechanism, but the manifestations of an emergent property embedded in the individual’s social interactions. We conceptualize different people as being depressive because the manifestations exhibited by them are sufficiently close to be conceptualized as identical in our social experience today. Because *Depression* is experienced as a reality today (by the depressed individuals and their social environment), we cannot consider that *Depression* is characterized solely by specific components (insomnia, fatigue, etc.) as is the case with Network Analysis, without taking into account a holistic view of *Depression* that considers *Depression* as an emergent property of individuals set in their social practice, an emergent property that causes perceptible manifestations (the components of Network Analysis models).

## Latent Variables and Emergent Properties

The approach considering psychological attributes as emergent properties overcomes the problem of the “black box” that can be seen as invalidating the use of latent variables (an issue raised by Van Der Maas et al., 2011). Based on a pragmatist-realist epistemology, we argue that Latent Variable models are efficient approaches to infer psychological attributes

### Emergence and Latent Variables

A psychological attribute is an emergent property of the individual embodied in social practices. We are in full agreement with Borsboom and Cramer (2013) who consider that in psychology we rarely have a clear relationship linking biological elements (a diseased organ diagnosed as such) to a psychological attribute. This does not mean that the psychological attribute does not exist, but that it exists without it being possible to define it biologically. The psychological attribute is perceptible not from an entity in the brain but from its manifestations. Categories are practical concepts based on real manifestations, concepts that reify different manifestations that seem similar in our social practice into a single category. The concept used to describe a psychological attribute appears coherent for our practical experience today. We therefore agree with Borsboom and Cramer (2013), for whom a psychological attribute is apprehended solely by its manifestations, because it is practical experience of the psychological attribute that determine its reality. It has to be inferred as a reality from its manifestations. Models using latent variables are precisely based on the perceptible manifestations of a psychological attribute. An understanding of psychological attributes as emergent properties gives legitimacy to models using latent variables in order to infer psychological attributes from their manifestations.

Latent variables thus become legitimate because the emergent properties can be conceptualized and analyzed on the sole basis of their manifestations, without exploring the underlying complex system. The latent variable relates to emergent properties, which have perceptible manifestations. The use of latent variables can therefore provide an operational framework for inferring psychological attributes. With latent variables, we have a reduction of the complexity of the system that generates the psychological attribute. We can indeed consider that latent variables, formalizing psychological attributes, reduce the complexity both conceptually and methodologically. Methodologically, we sidestep the complexity, and there is thus an irreversible loss of information (no analysis of the complexity upstream of the psychological attribute). Conceptually, however, there is a gain in information by clarification of the emergent property.

### The Common Cause

The central criticism in the Network Analysis literature against Latent Variable models is above all the denial that there is a “common cause” of the manifestations/components observed. The Network Analysis literature has pointed out that, for Latent Variable models, manifestations must be interchangeable, a framework that requires local independence. As Fried (2015) considers, this assumption is implausible for depression for example. *Insomnia* may cause *Fatigue*, which in turn can trigger *Loss of interest*. Depressive symptoms directly influence each other, and the symptoms are not equivalent or interchangeable (Fried, 2015). Moreover, there is a lack of homogeneity in depressive syndromes because symptoms differ from one individual to another. Consequently, for Fried there is no common cause and depression is not a natural category.

This raises the issue of how psychological attributes are conceptualized with Network Analysis. If Network Analysis links some components to a specific psychological attribute and not others, it is because there is a sort of homogeneity in correlated behaviors (components) categorized as relating to one and the same attribute. It is one thing to consider the potential relational structure between components, as in Network Analysis; it is another to reduce the attribute to this component relationship without considering a more holistic psychological attribute. Considering that there is a “common cause” does not mean that there is a localizable physical entity, but that the correlations between components result from a common underlying mechanism that generates them and explains the correlated behaviors. This is an abductive procedure inferring a common cause to be a reality, it does not consider the theory as *true*, but as *acceptable* (Haig, 2005a,b). Abduction was proposed by Peirce (1905) who developed a pragmatist epistemology, and abduction is not based on the supposition that truth about an independent reality can be irrefutably established, but on the idea we have to find the best explanation given the limits of our practice (Baggini and Fosl, 2010).

In the line of Peirce, pragmatism-realism seems to us an efficient epistemological framework for psychology, a *coherentist* approach (Rorty, 2000). Psychological attributes underpin the social existence of individuals. We conceptualize similar manifestations on the part of different individuals as relating to a particular psychological attribute. The concepts thus used make it possible to describe/explain internal states that give rise to observable behaviors. The categories used in psychology (psychological concepts) concern realities that exist by social consensus arising in a social framework, they are observer-dependent. Psychological attributes are classified according to their manifestations and their social communication function/regulation. These categories are linked to realities, but derived from human experience and social interactions. This conceptualization of psychological attributes amounts to considering them as inter-subjective realities, which we can consider as a *common cause for us*.

The issues of the common cause and local independence raised by Network Analysis confuse two issues. Latent Variable models assume the local independence of manifestations, which contradicts the interrelations between manifestations. This raises a methodological issue, and we will return to this problem in the next section. But considering that manifestations interact is, ontologically, not incompatible with the consideration that there may be a common cause to explain these correlated manifestations. We can consider that manifestations have a common cause and that they interact.

### Latent Variables Combined with Network Analysis

Network Analysis raises the issue of the local independence of manifestations required by Latent Variable approaches. As explained before, considering that manifestations reveal an underlying mechanism (a common cause) is not in contradiction with the hypothesis that manifestations can interact. We consider that Latent Variables and Network Analysis are not mutually exclusive approaches, they are complementary. In fact, some authors who previously advocated Network Analysis rather than Latent Variable models seem to be returning to Latent Variable models. Epskamp et al. (2017, p. 2) today consider that “network modeling and latent variable modeling can complement — rather than exclude — one another… we think the assumption of no underlying latent traits … may often be too strict.” The new approach proposed by Epskamp et al. (2017) seems an interesting approach combining the Latent Variable approach and Network Analysis. It retains the notion of the latent variable and thus views psychological attributes as realities (common causes of manifestations). Psychological attributes can be considered to be measured by manifest variables in reflective manner, as in classic *Structural Equation Modeling* (SEM). This approach estimates not the causal links (as in a regression), but rather the network of latent variables. This network is estimated by pairwise interactions (Epskamp et al., 2017). It has no equivalent model, unlike classic SEM in which equivalent models are possible (Lee and Hershberger, 1990; MacCallum and Browne, 1993; Raykov and Marcoulides, 2007). With Latent Network approach, the objective is not to estimate *causal relationships* between latent variables, but *interactions* between these latent variables. Consequently the model can generate a graph that is not a-cyclic because “much psychological behavior can be assumed to have at least some cyclic and complex behavior and feedback” (Epskamp et al., 2017, p. 10). After modeling the network of latent variables, we can model the network of manifest variable residuals. This approach is identical to a Network Analysis, but based on residuals from the latent network. The local independence of manifest variables is avoided because we can have interactions between manifest variables that are independent from the latent variables.

This new approach is a major opening in response to the issue of classic SEM versus Network Analysis: we retain latent variables and we introduce the possibility of cyclic relations between manifestations or latent variables. But we consider that the Latent Network models (and the associated Residual Network models) raise epistemological issues. With Latent Network models, latent variables need to be considered in a dynamic perspective because these models place the individual in a loop effect impacted by the environment. As a result, psychological attributes emerge from “a network of interacting psychological, sociological and biological components” (Epskamp et al., 2017, p. 2). This dynamic perspective, where individuals interact with their environment, suggests that concepts and models relating to psychological attributes need a pragmatist-realist epistemology.

### Example of Depression

If we return to the example of *Depression*, we agree that a Network Analysis approach could improve knowledge on the subject of *Depression*. We consider that Network Analysis, by focusing on components and the trajectories of these components, opens the way to innovating analyses of *Depression* that break with a normative point of view (see Bringmann et al., 2015; van Borkulo et al., 2015; Boschloo et al., 2016; Fried et al., 2016). It is not a contradiction to consider *Depression* as an inter-subjective reality. More explicitly, using a Network Analysis model is not in contradiction with the idea that there is a common mechanism underlying the components integrated into the model for each individual. This underlying mechanism is not a natural category, and from this point of view Network Analysis really is an improvement. *Depression* is a practical category used (abductively) to explain correlated manifestations/components – a holistic, embedded conceptualization of *Depression*.

We are in agreement with Fried (2015), who considers that *Depression* is today a fuzzy category that overlaps with various other syndromes as they are at present categorized. The “interactive genres” or relational categories of psychological attributes (Hacking, 1998, 2000) like *Depression* suggest that we need to reappraise the concepts and the measures of psychological attributes. The criticism against the categorization of *Major Depressive Disorder* by the DSM by different authors (Fried et al., 2014; Cramer et al., 2016; Nuijten et al., 2016) certainly points the way to reappraising *Depression*. In the future, we may come to consider *Depression* as a multiform category (as is already the case with certain authors), possibly with different sub-categories. We may also come to categorize *Depression* by way of different concepts without using the term of *Depression* – just as *Melancholia* was removed from the vocabulary of psychopathology.

As we explained, a pragmatist-realist framework of depression does not go against a certain reality of *Depression*. But, pragmatism-realism denies that there is any such thing as “true” *Depression*, a reality independent from the social praxis of this illness. If we categorize individuals in a given category *Depression*, this results from a sort of homogeneity in the manifestations in some individuals compared to others, and from a certain homogeneity in our social practices (and in our practical categorizations). Thus, there is still today a “reality for us” in the notion that individuals categorized as *Major Depressive Disorder* really are in *Depression*. If we consider that *Depression* is an outdated concept, as appears to be the case with the Network Analysis literature, we need to develop new concepts to categorize these different illnesses, as Hacking explains, shifting to other psychological attributes (Hacking, 1998).

## Conclusion

Latent Variable models are criticized by some authors because we cannot generally find any entity in the brain that causes the manifestations we are exploring. Network Analysis is proposed as a new approach to infer psychological attributes. We consider that Network Analysis has epistemological problems. Network Analysis has attempted to link complex processes and emergent properties. If we want to analyze the emerging properties of a complex system, we need to conceptualize and model on the level of the emergent property, without recourse to the network that generated it. Because psychological attributes are properties relating to an individual embedded in social interactions, they can be considered solely as human manifestations. As Hacking (1998, 2000) explained, psychological attributes are *moving* categories because they are related to social interactions, and thus dependent on a social matrix to conceptualize these manifestations. The term moving is not to be understood in the sense that there is no equilibrium for an individual. A psychological attribute characterizes mental equilibrium in the sense that it characterizes an emergent property of individuals within their social practice. The categories used are nevertheless a practical formalization strung between reality and social construction. We need to reappraise the concepts in relation to their context and the issues they raise, which generate shifts in categories over time. Categories formalized by psychology possess relevance only at the time of their use, and they should not be considered as valid truths at any time and in any place.

Latent Variables models therefore seem relevant for the analysis of psychological attributes, considered as emergent properties of the individual, a common cause of the manifestations. We should not, however, seek to integrate the complexity of the underlying processes. The Latent Variable approach does nevertheless consider psychological attributes as realities belonging to individuals within their social praxis, which are thus only perceptible from social manifestations. We therefore consider that the models using latent variables are difficult to avoid for anyone who sets out to analyze psychological attributes as social realities. Because psychological attributes are non-reducible emergent properties, and are dependent on the environment, this need to resort to latent variables is not consistent with an epistemology that considers psychological attributes as physical entities in the brain independent from human praxis (*biological realism*, Lloyd, 2010).

The Network Analysis approach has the failing of aiming to model what is complex, thus losing sight of the entity that is to be modeled: the psychological attribute. But the interest of this approach is that it integrates the interdependence of individuals with their environment by integrating interrelated events, and this is a real breakthrough in the academic literature, which all too often considers psychological attributes as isolated biological objects. Network Analysis can serve to improve Latent Variable models, and it also entails a criticism of the inertia and conformism that drive most models using latent variables (Sijtsma, 2012). *Latent Network Models* and *Network Residuals*, as proposed by Epskamp et al. (2017), are certainly a promising new way to infer psychological attributes, combining latent attributes and dynamic systems of manifestations. However, this methodology places psychological attributes in an inter-subjective approach (Allen and Williams, 2011), which needs to find a more appropriate epistemological framework: one that is both pragmatist and realist.

## Author Contributions

HG developing conceptual discussion and wrote the first draft. BF developing conceptual discussion. J-LK developing conceptual discussion.

## Conflict of Interest Statement

The authors declare that the research was conducted in the absence of any commercial or financial relationships that could be construed as a potential conflict of interest.

## Footnotes

1 We use the term “component” as a generic term for “observables,” “symptoms,” “indicators,” etc. used in the Network Analysis literature.

2 We do not discuss here the issue of the measurement psychological attributes (Michell, 1999; Sherry, 2011; Markus and Borsboom, 2012; Sijtsma, 2012). In our discussion, measures can be quantitative, ordinal or qualitative.
